# Non-Enzymatic Depurination of Nucleic Acids: Factors and Mechanisms

**DOI:** 10.1371/journal.pone.0115950

**Published:** 2014-12-29

**Authors:** Ran An, Yu Jia, Baihui Wan, Yanfang Zhang, Ping Dong, Jing Li, Xingguo Liang

**Affiliations:** College of Food Science and Engineering, Ocean University of China, Qingdao, 266003, China; Southern Illinois University School of Medicine, United States of America

## Abstract

Depurination has attracted considerable attention since a long time for it is closely related to the damage and repair of nucleic acids. In the present study, depurination using a pool of 30-nt short DNA pieces with various sequences at diverse pH values was analyzed by High Performance Liquid Chromatography (HPLC). Kinetic analysis results showed that non-enzymatic depurination of oligodeoxynucleotides exhibited typical first-order kinetics, and its temperature dependence obeyed Arrhenius’ law very well. Our results also clearly showed that the linear relationship between the logarithms of rate constants and pH values had a salient point around pH 2.5. Interestingly and unexpectedly, depurination depended greatly on the DNA sequences. The depurination of poly (dA) was found to be extremely slow, and thymine rich sequences depurinated faster than other sequences. These results could be explained to some extent by the protonation of nucleotide bases. Moreover, two equations were obtained based on our data for predicting the rate of depurination under various conditions. These results provide basic data for gene mutagenesis and nucleic acids metabolism in acidic gastric juice and some acidic organelles, and may also help to rectify some misconceptions about depurination.

## Introduction

Depurination, the release of purine bases from nucleic acids by the hydrolysis of N-glycosidic bonds, has aroused considerable interest for a long time because of its close relationship with mutation and repair of nucleic acids. At apurinic sites caused by depurination, the covalent structure of DNA becomes more susceptible to damage, which induces spontaneous mutagenesis, carcinogenesis and cellular aging [1]–[8]. It has been estimated that approximately 2,000–10,000 DNA purine bases are released in each human cell every day due to hydrolytic depurination [9], [10]. In addition, Zhang *et al.* recently found that nucleic acids in food could be absorbed by alimentary systems and they regulated the expression of target genes in mammals [11]. On the other hand, efficient depurination of nucleic acids may occur in acidic gastric juice and some acidic organelles (such as lysosomes), which then shows effects on digestion and assimilation of nucleic acids [12]. Until now, very few studies have analyzed the details of the digestion of nucleic acids in the stomach.

Although depurination has been investigated for decades, there are still some unsolved controversies. For example, the depurination of mononucleotides and short oligonucleotides has been shown to be first-order reactions [13]–[15], but long calf thymus DNA depurinated quite slowly for the first several hours, deviating from the first-order profile [16]. Different opinions were also raised on whether there was a simple linear relationship between pH and the logarithm of depurination rate constants [17]–[20]. In addition, it is still not clear whether depurination depends on sequences, although some reports have claimed that depurination was not markedly dependent on DNA sequences [10], [14]. These inconsistent results may be caused by data lacking accuracy and reliability due to some imprecise separating and detecting methods, such as dialysis and thin-layer chromatography. Furthermore, because the published data are scattered and sometimes incomprehensive, it is difficult to predict the degree of depurination under certain conditions.

In this study, we designed and used a pool of 30-nt-long short oligodeoxynucleotides (ODNs) with various sequences for depurination and provided some answers to the above controversies by systematically investigating factors of depurination including pH, salts and secondary structure (duplex or single stranded). The effect of DNA sequences on depurination was also studied for the first time. In addition, prediction of depurination degree under various conditions was realized based on two equations we obtained.

## Materials and Methods

### Materials

All ODNs used in this study were ordered from Integrated DNA Technologies, Inc. (Coralville, IA, USA) and dissolved in sterile Milli-Q water to 100 µM for stock. M13mp18 single-stranded DNA (M13 ssDNA, 250 µg/mL), M13mp18 RF I DNA (M13 dsDNA, 100 µg/mL) and Lambda DNA (300 µg/mL) were purchased from New England Biolabs, Inc. (Beverly, MA, USA). Salmon sperm DNA (Sigma-Aldrich, WI, USA) was dissolved in sterile Milli-Q water to 300 µg/mL and used as the substrate for depurination. Nucleotide bases, including adenine, guanine, thymine, cytosine and uracil (Sigma-Aldrich, WI, USA) were used as the standard substances for HPLC analysis. They were dissolved in sterile Milli-Q water to 200 µg/mL, respectively, and diluted to the final concentration of 20 µg/mL in mixed samples.

### Melting temperature (*T*_m_) measurement

The melting curve of M13 dsDNA at pH 5.1 was obtained by measuring the change in absorbance at 260 nm versus temperature with a UV-1800 spectrophotometer equipped with a temperature-controlling device (Shimadzu Co., Kyoto, Japan) under the following conditions: 50 mM phosphate sodium buffer (pH 5.1) and 12 µg/mL M13 dsDNA. To minimize the depurination of M13 dsDNA at pH 5.1 during the *T*_m_ measurement, only the heating curve was measured. The temperature ramp was 1.0°C/min.

### Depurination of nucleic acids

In the experiments, DNA substrates for depurination (to the final concentration of 10 µM of ODNs, 25 µg/mL of M13 ssDNA, M13 dsDNA, Salmon sperm DNA or Lambda DNA during depurination) and uracil, serving as the internal standard (to the final concentration of 37.5 µM), were added to the following buffers: aqueous solution of 50 mM sodium phosphate with various pH from 0.5 to 7.1 or acidic solutions (pH 1.4 or pH 5.9) of NaCl (5−1000 mM) or MgCl_2_ (1−100 mM). Depurination was performed at a constant temperature on TC-5000 thermal cycler (Techne, Staffordshire, UK). To terminate the reaction, the pH values of aliquots were adjusted to 7.0–8.0 by NaOH solution in various concentrations.

### HPLC analysis of reaction products

A 230II type of HPLC system (Elite, Dalian, China) and YMC C18 column (250×4.6 mm, 5 µm; YMC, Kyoto, Japan) were used for depurination analysis. The following HPLC conditions were used to separate free purines: a linear gradient from 4% to 14% (20 min) acetonitrile in the water containing 50 mM ammonium formate; a flow rate of 0.5 mL/min; detection at 260 nm; and 10 µL of loaded reaction solution.

For precise quantification, uracil was used as the internal standard. Release of purines in each experiment under strong conditions (pH 1.6 and 100°C for 1 h; with the same concentration of uracil as the internal standard) was served as the total purines for calculating the percentage of depurination. The percentage of released adenine or guanine (P_t_) after a period of time (t) can be calculated by this formula: P_t_ = S_P_×S_U0_/(S_U_×S_P0_), where S_P_ and S_U_ are the peak areas for removed purines (adenine or guanine) and uracil (internal standard) in the sample groups for analysis; S_P0_ and S_U0_ are the peak areas under strong conditions for removing purines totally. The rate constants (*k*) of depurination were obtained when the percentages of depurination were less than 20% and calculated by averaging the data of released adenine and guanine.

## Results

### Depurination of a pool of 30-nt-long ODNs with various sequences

Considering that natural long-chain DNA tends to form sediments at low pH and is difficult to be analyzed on a HPLC system directly, we used a pool of 30-nt-long ODNs with various sequences (N30) to describe the general features of non-enzymatic depurination. Theoretically, there are approximately 10^18^ types of 30-nt-long sequences in the pool, which were synthesized on a DNA synthesizer with mixed phosphoramidite monomers (IDT, IA, USA). These sequences can comprehensively represent most genome sequences for depurination, though they are only 30-nt long. Another merit to using this pool is that almost no duplex can form, especially under acidic conditions, because only one 30-nt-long molecule for each sequence is present. The dynamic analysis becomes more accurate without the effect of sequence variation and the difference in depurination between double-stranded (dsDNA) and single-stranded DNA (ssDNA) can also be minimized.

The products of depurination from N30 were completely separated and quantitatively analyzed by HPLC using uracil as the internal standard (Figure S1). Each depurination experiment was performed 3 times, and the error was further minimized by using the complete depurination samples (at pH 1.6, 100°C for 1 h) as the reference of the total amounts of purines for calculating the percentage of depurination.

**Figure 1:**
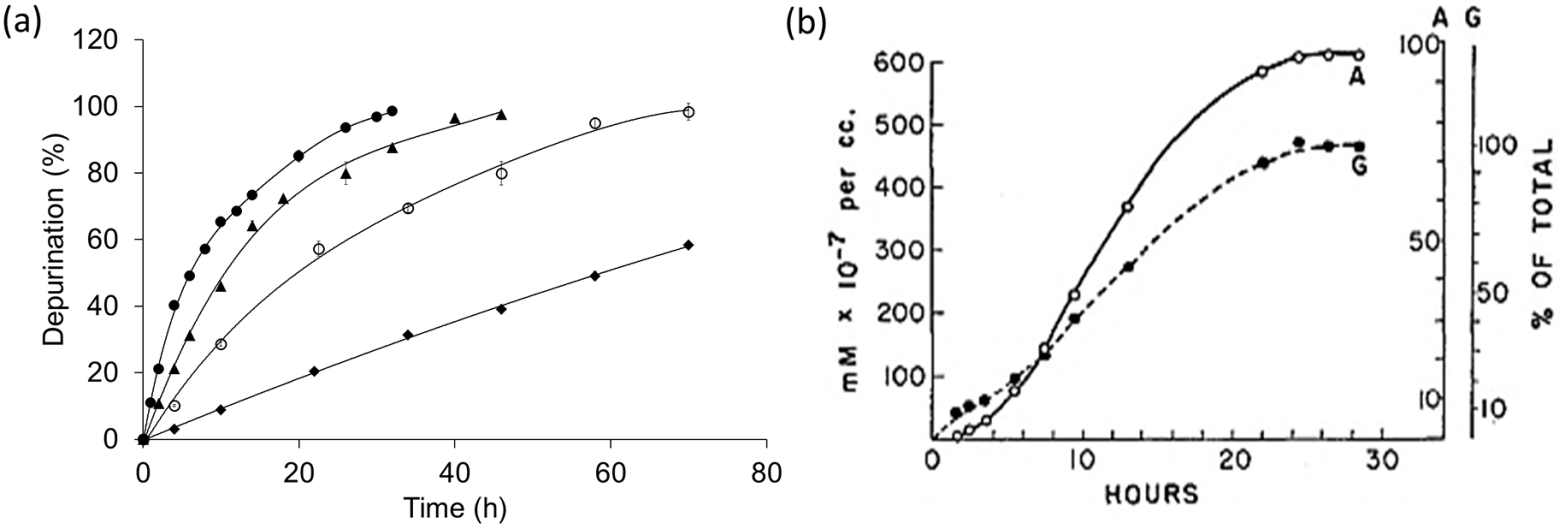
Time courses of non-enzymatic depurination. (a) Quantitative analysis of purines released from N30 at 37°C. (•) pH 1.6; (▴) pH 2.0; (○) pH 2.5; (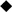) pH 3.0. Reaction buffers for depurination contained 50 mM sodium phosphate. Samples were prepared by collecting aliquots of the solution in each time period. The percentages of depurination were obtained by averaging the percentages of the release of adenine and guanine. The plot shows the average of three individual experiments. (b) Depurination of calf thymus DNA at 37°C and pH 1.6 reported by Tamm *et al*. [16]. (○) Adenine; (•) Guanine.

At first, we checked the time courses of depurination from N30 under various pH values. As shown in Figure 1a, the initial rates of the depurination reactions remained constant, and the time courses obeyed the model of first-order kinetics perfectly. Thus, the rate constants (*k*) could be obtained from the above time courses. We also checked the depurination from DNA in various concentrations from 0.5 µM to 50 µM and found that the rate constants were independent of DNA concentration, which was also consistent with the first-order kinetics (data not shown). It was reported that an abnormal slowness was observed during the first several hours of depurination of long calf thymus DNA (Figure 1b), which was different from our results [16].

The rate constants of depurination measured at various pH values are shown in Table 1. Certainly, depurination depended greatly on the reaction temperature and was much faster under lower pH conditions. For pH values above 5.0, detection of depurination at 37°C became difficult due to the extremely low speed. After incubation at 37°C for 33 d, N30 released 5.8%, 0.6% and 0.1% purines at pH 5.1, pH 6.1 and pH 7.1, respectively (Figure S2). The difference in depurination between adenine and guanine was not obvious, and only a change of 3–25% was observed (Table 1).

**Table 1:** Rate constants (*k*, s^−1^) of depurination from N30 at 37°C.

pH	Guanine (*k*, s^−1^)	Adenine (*k*, s^−1^)
1.0	9.0×10^−5^	9.7×10^−5^
1.6	2.9×10^−5^	3.0×10^−5^
2.0	1.4×10^−5^	1.6×10^−5^
2.5	7.2×10^−6^	7.8×10^−6^
3.0	2.0×10^−6^	2.5×10^−6^
3.7	4.1×10^−7^	5.0×10^−7^
4.1	2.1×10^−7^	2.4×10^−7^
5.1	2.4×10^−8^	1.9×10^−8^
6.1	2.4×10^−9^	2.0×10^−9^
7.1	2.2×10^−10^	2.5×10^−10^

### Factors for depurination

The pH values, sequences, ion strength and temperature, as well as duplex state or not, affect the depurination speed. Until now, there are still some controversies and uncertainties about some of these factors. For clarifying these questions and realizing the prediction of depurination under various conditions, we checked all these factors in detail.

#### Effect of pH on depurination

**Figure 2:**
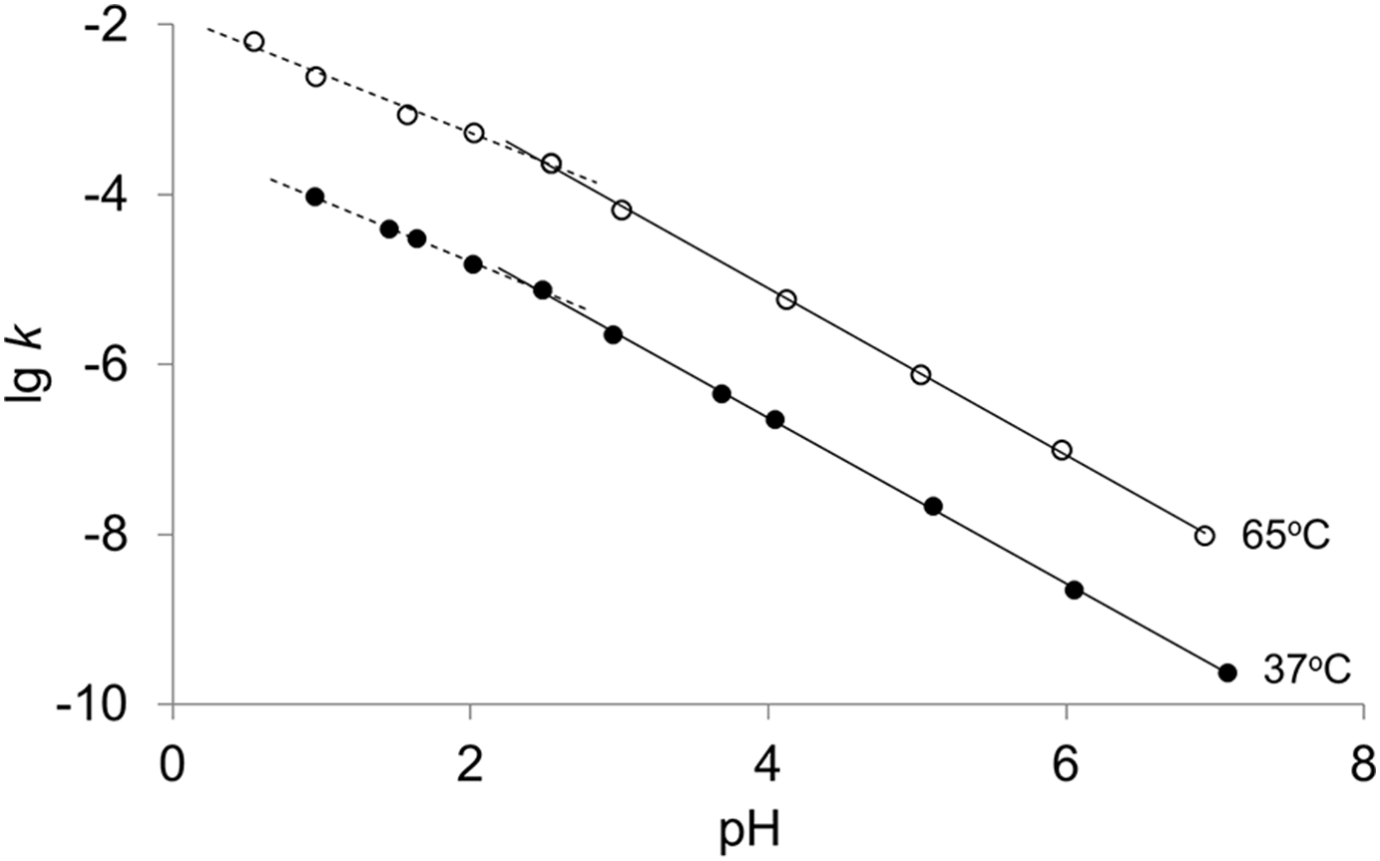
Plots of rate constants (*k*, s^−1^) for the depurination of N30 as a function of pH. (•) 37°C, the slopes are −0.721 at pH 1.0−2.5 and −0.975 at pH 2.5−7.1; (○) 65°C, the slopes are −0.694 at pH 0.5−2.5 and −0.983 at pH 2.5−7.1.

To solve the controversy about pH dependence of depurination, especially about whether the pH profile is a straight line [17]–[20], we checked carefully the rate constants of N30 at a wide range of pH from 0.5 to 7.1. As concluded previously, the depurination rates of adenine and guanine were very close and so the average depurination rates of adenine and guanine were used here. As shown in Figure 2, the logarithm of depurination rate constants (lg *k*) basically showed a linear relationship with pH at either 37°C or 65°C. Interestingly, our results clearly showed that there was an inflection point at a pH of approximately 2.5 for both reaction temperatures. At 37°C, for example, the absolute value of the slope below pH 2.5 was obtained as 0.721, whereas it increased to 0.975 above pH 2.5, indicating that the effect of pH on depurination was weakened at a pH lower than 2.5.

#### Effect of reaction temperature on depurination

**Figure 3:**
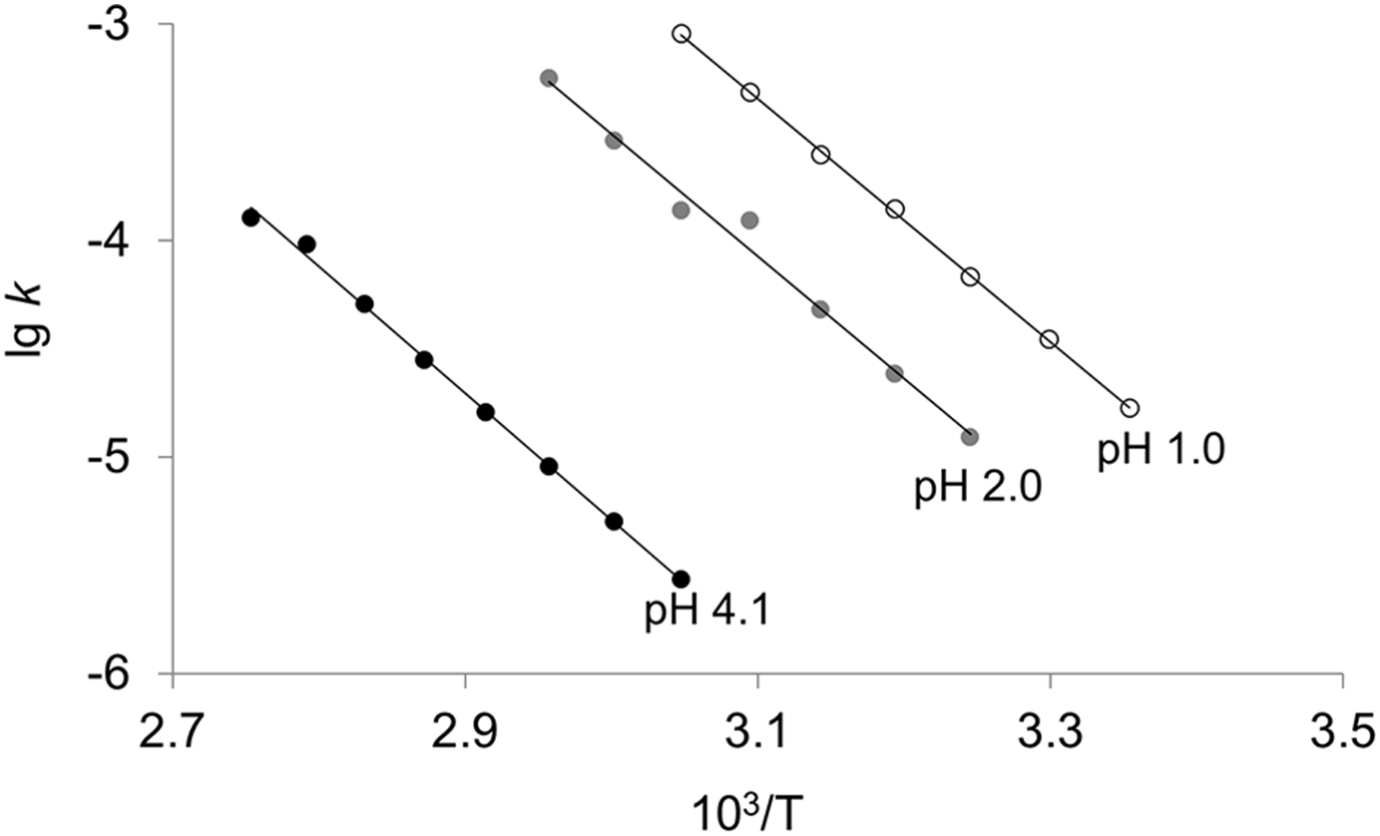
Arrhenius plot for depurination from N30 at different pH values. Rate constants (*k*, s^−1^) of depurination were a function of absolute temperature (T). (○) pH 1.0; (•) pH 2.0; (•) pH 4.1.

Reaction temperature may not only affect the depurination reaction itself but also may affect the secondary structure of DNA, especially at a higher pH. Considering that the dynamics of depurination at pH<2.5 were different from those at pH>2.5, the effect of temperature was investigated at pH 1.0, pH 2.0 and pH 4.1. As shown in Figure 3, the temperature dependence of depurination obeyed Arrhenius’ law very well. The activation energies (*E_a_*) and frequency factors (*A_r_*) were obtained from the slopes and intercepts. In terms of the activation energy, little difference was observed for depurination reactions at pH 1.0 (*E_a_* = 107.5 kJ/mol)), pH 2.0 (*E_a_* = 108.2 kJ/mol) and pH 4.1 (*E_a_* = 112.0 kJ/mol). On the other hand, the value of the frequency factor at pH 1.0 (*A_r_* = 1.2×10^14^) was 4.3-fold larger than that at pH 2.0, and 63-fold larger than that at pH 4.1, indicating that the observed distinction in depurination rates between different pH values was mainly caused by the difference in the frequency factors.

#### Effect of salts on depurination

**Figure 4:**
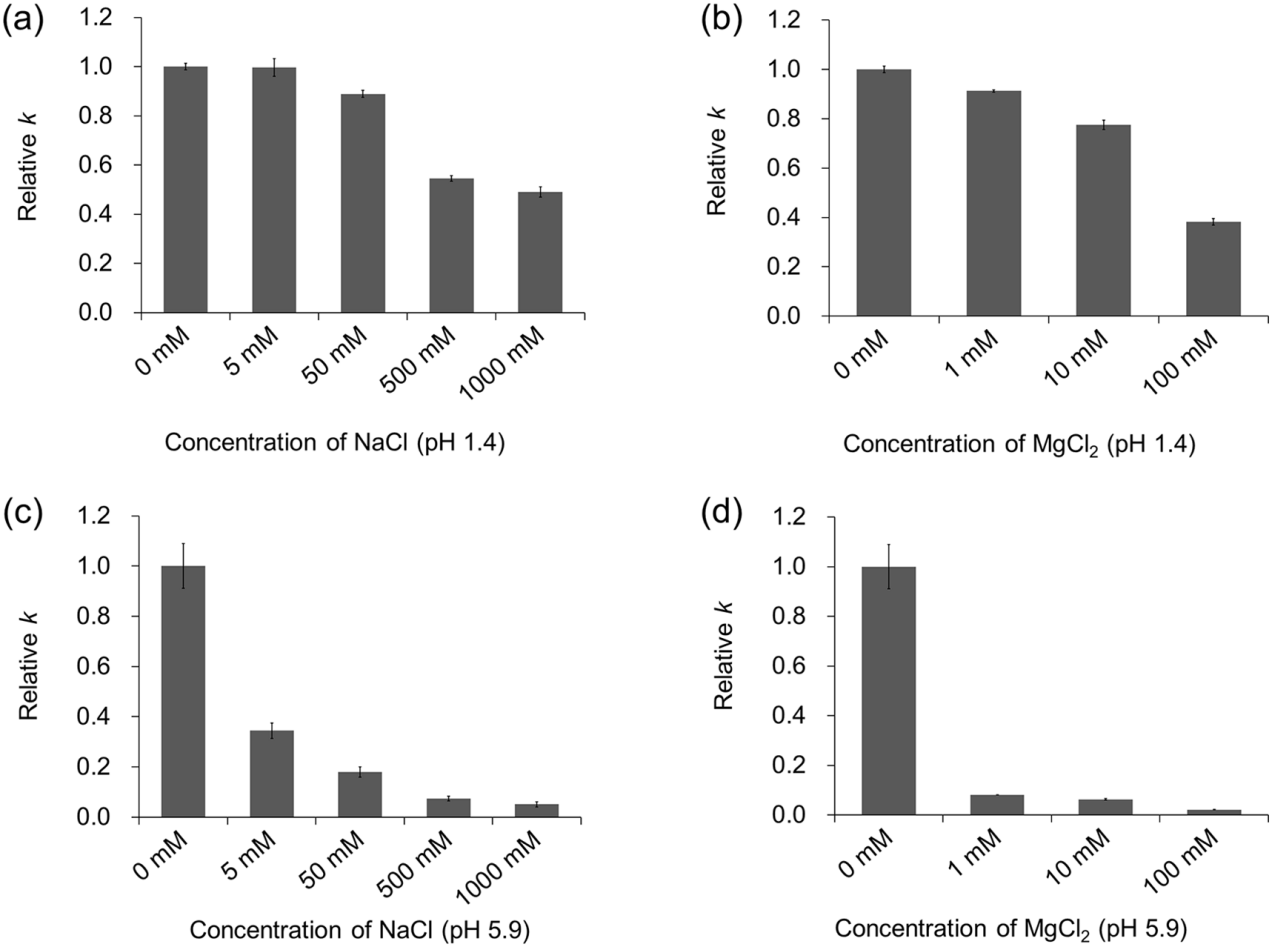
The effect of salts on depurination from N30. (a) Depurination in the NaCl buffer (pH 1.4); (b) Depurination in the MgCl_2_ buffer (pH 1.4); (c) Depurination in the NaCl buffer (pH 5.9); (d) Depurination in the MgCl_2_ buffer (pH 5.9). The pH values of the buffers were adjusted to 1.4 (±0.1) or 5.9 (±0.2) with an aqueous solution of HCl. The reaction was performed for 60 min at 37°C (pH 1.4) or 24 h at 80°C (pH 5.9) to limit the percentages of depurination less than 20%. In each chart, the depurination rate constant of N30 in the pure HCl buffer was served as the reference.

Salts may affect depurination rates due to their neutralization of negative charges on phosphate groups. In addition, considering that salts intake can change the salt concentration in gastric juice, figuring out the salt effect is of great significance on depurination both *in vivo* and *in vitro*. Figure 4 shows the effect of salt concentration on the rate of depurination. For all the conditions used in our study, depurination was suppressed by salts to different degrees, and the suppression degrees of salts were affected significantly by pH values. In the case of pH 1.4, for example, the rate constant (*k*) for 50 mM NaCl decreased by 10% compared to that for 5 mM NaCl (Figure 4a). When N30 was incubated at pH 5.9 (Figure 4c), depurination for 5 mM NaCl was found to be twice as fast as that for 50 mM NaCl, indicating that the suppression ability of salts on depurination rose dramatically with the increase in pH values. The same phenomenon was observed for the solution of MgCl_2_. In addition, compared with depurination in the solution of NaCl and MgCl_2_ at pH 5.9 (Figs. 4c and 4d), the suppression effect of 1 mM Mg^2+^ was basically similar to that of 500 mM Na^+^. Therefore, the inhibition of Mg^2+^ on depurination was much stronger than that of Na^+^.

#### Prediction of depurination rates under various conditions

Summarizing the above results, we can predict the depurination rates under certain conditions. As is shown in equation (1) and (2), the rate constants (*k*) can be estimated at a given pH and temperature:
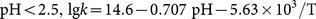
(1)

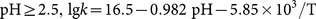
(2)


In the equations above, *k* is in s^−1^ and T is the absolute temperature during depurination. It is worth mentioning that these predicting formulas were obtained when 50 mM sodium phosphate was used as reaction buffer. As the effect of salts is much smaller than that of pH and temperature, the depurination rate of DNA can be successfully predicted with our formulas as long as the reaction conditions are set.

### Dependence of depurination rates on sequences

**Figure 5:**
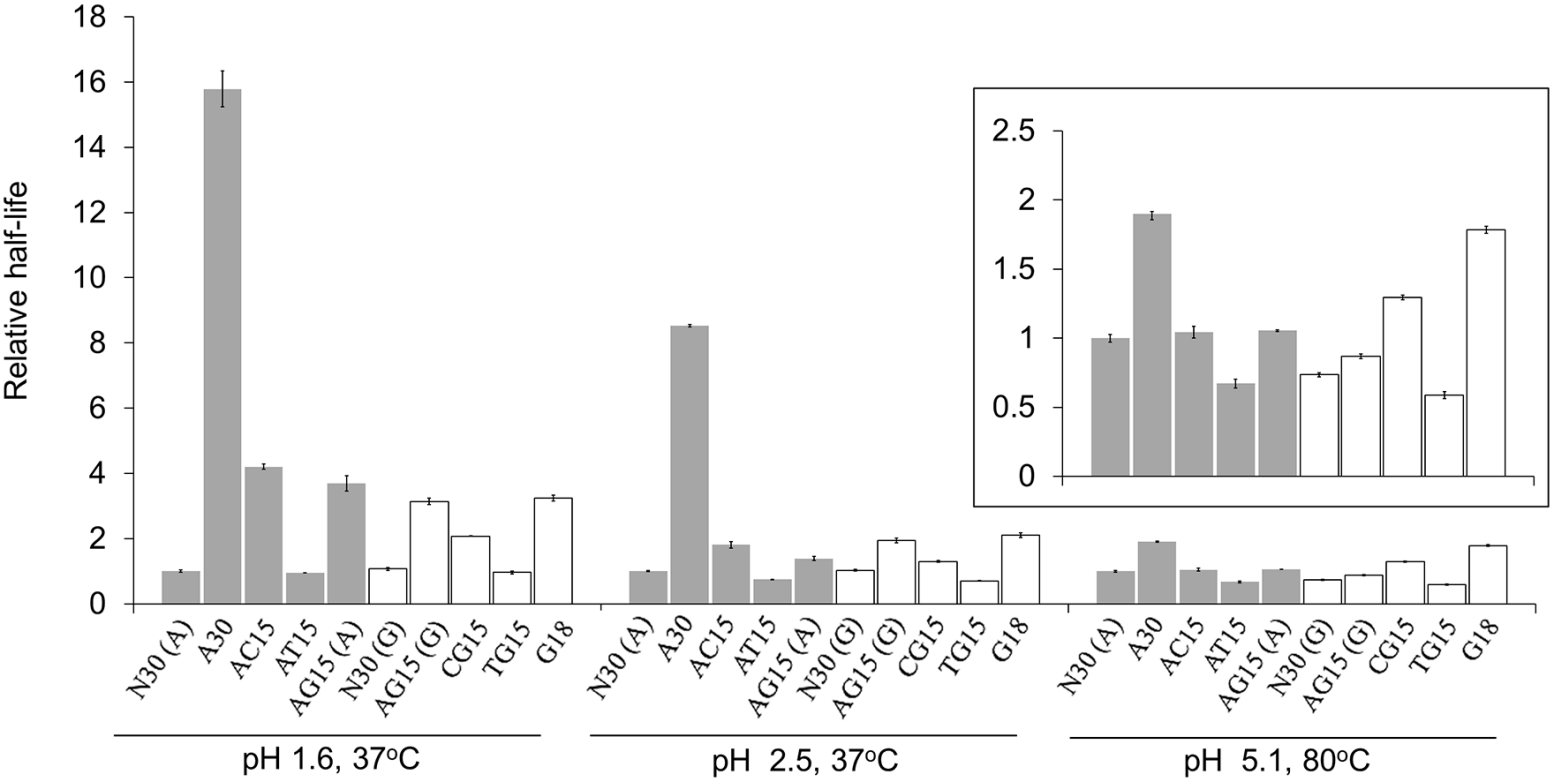
Difference of various sequences for depurination. (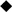) Adenine; (□) Guanine. In each reaction condition, the half-lives of N30 (depurination of adenine) were served as the references. The sequences used for depurination are listed in Table 2. The insert is the enlarged view at pH 5.1, 80°C.

Until now, the effect of DNA sequences on depurination has not been clarified because it is difficult to study this effect with natural DNA. Here, several simple repetitive sequences (Table 2) were used to amplify the sequence effect. The relative half-lives for various sequences, with the half-life of N30 (depurination of adenine) as the reference, are shown in Figure 5. Interestingly, depurination rates changed dramatically with different sequences. In terms of adenine, the order of depurination rates of these sequences at pH 1.6 was AT15≥N30>AG15>AC15>A30, and the order of guanine was TG15≥N30>CG15>AG15≥G18. The orders indicated that sequences without thymine bases depurinated much slower than N30, especially for A30. For sequences with thymine, such as AT15 and GT15, depurination was much faster. At pH 1.6, for example, the half-life of A30 (t_1/2_ = 97 h) was 16.8-fold longer than that of AT15 (Figure 5). The phenomenon that thymine accelerating the depurination rate could also be observed when trinucleotide repetitive sequences with 2/3 thymine were used for depurination (Table S1).

**Table 2:** DNA sequences used for depurination.

Name	Sequence (5′→3′)
N30	NNNNN NNNNN NNNNN NNNNN NNNNN NNNNN^a^
A30	AAAAA AAAAA AAAAA AAAAA AAAAA AAAAA
AC15	ACACA CACAC ACACA CACAC ACACA CACAC
AT15	ATATA TATAT ATATA TATAT ATATA TATAT
AG15	AGAGA GAGAG AGAGA GAGAG AGAGA GAGAG
TG15	TGTGT GTGTG TGTGT GTGTG TGTGT GTGTG
CG15	CGCGC GCGCG CGCGC GCGCG CGCGC GCGCG
G18	GGGGG GGGGG GGGGG GGG

a N = A, T, C or G.

More interestingly, the diversity of depurination rates among different sequences decreased gradually with the rise of pH (Figure 5). In the case of pH 2.5 at 37°C, for example, the half-life of A30 (t_1/2_ = 230 h) was 11.4 times longer than that of AT15. At pH 5.1 and 80°C, however, the difference was only 2.8 times. It is worth pointing out that some researchers reported that depurination was not markedly dependent on the DNA sequences [10], [14], most likely because their experiments were carried out at pH values above 5.0. As we described previously, the influence of incubating temperature on the difference among these sequences can be ignored.

### Difference between single-stranded (ssDNA) and double-stranded DNA (dsDNA)

To determine the difference in depurination rate between dsDNA and ssDNA, DNA from bacteriophage M13mp18 in single-stranded (M13 ssDNA) and double stranded (M13 dsDNA) forms was applied for depurination. The use of M13 ssDNA can ensure almost no duplex forms during depurination, which is better than using denatured DNA to check the depurination rate of ssDNA [10].

At first, we checked the temperature range for M13 dsDNA to maintain the duplex form at pH 5.1. From the melting curve we measured (Figure S3), the *T*_m_ at pH 5.1 was determined as 82.5°C, and M13 dsDNA could keep the duplex form below 70°C. Considering that depurination also decreased the *T*_m_ of a DNA duplex, we selected 60°C as the reaction temperature.

**Figure 6:**
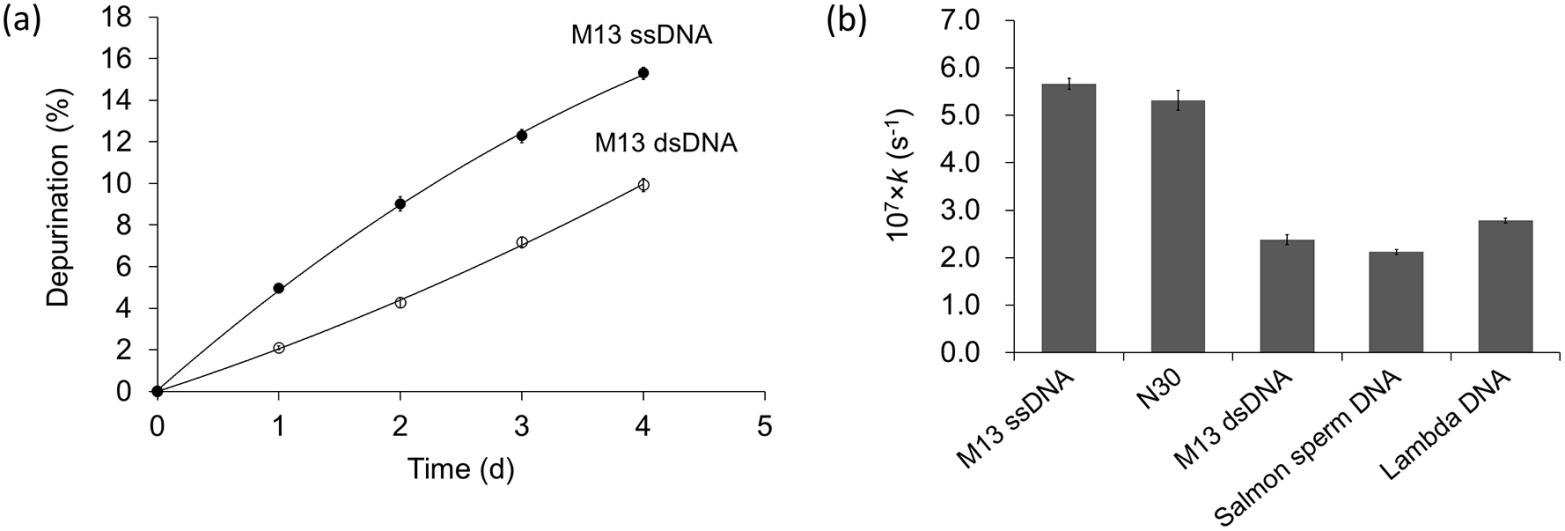
Difference between single-stranded and double-stranded DNA for depurination at pH 5.1, 60°C. (a) Time courses of depurination of M13 ssDNA and dsDNA. (○) M13 dsDNA; (•) M13 ssDNA. (b) The rate constants (*k*) of several kinds of DNA including ssDNA (M13 ssDNA and N30) and dsDNA (M13 dsDNA, Salmon sperm DNA and Lambda DNA). *T*_m_ analysis showed that M13 dsDNA kept duplex form below 70°C at pH 5.1.

It is shown clearly in Figure 6a that DNA depurinated slower in the duplex structure. After incubation for 4 d, M13 ssDNA released 15.3% of purines, while M13 dsDNA only lost 9.9%. The rate constant of M13 ssDNA (*k* = 5.7×10^−7 ^s^−1^) was 2.4 times higher than that of double-stranded form.

In addition, other DNA sequences including N30, Lambda and Salmon sperm DNA were also used to confirm the difference between ssDNA and dsDNA (Figure 6b). The rate constants of depurination from N30 (ssDNA) were 2.5 and 1.9 folds larger than that from Salmon sperm DNA and Lambda DNA (dsDNA), respectively.

## Discussion

In this study, a pool of 30-nt-long ODNs with various sequences (N30) was used as the substrate for depurination under various conditions. It was reported that some special sequences with a potential to form a stem-loop structure (a 5′-G(T/A)GG-3′ loop and 5′-T•A-3′ or 5′-G•C-3′ as the first closing base pair) performed self-catalyzed, site-specific depurination, which was 4–5 orders of magnitude faster than the background spontaneous depurination under physiological conditions [21], [22]. However, only a tiny percentage of oligomers (∼0.006%) are present in the pool of 30-nt-long ODNs, and the influence on our depurination results can be ignored. On the other hand, the sequences for self-catalytic depurination are widely distributed in genomes across the phyla [23]. The presence of these sequences in the substrate of N30 for depurination is beneficial to represent the general feature of the depurination of genome sequences.

Through quantitative analysis of the depurination from N30, several controversies on depurination were clarified. First of all, as shown in our data, non-enzymatic depurination of ODNs was a typical first-order reaction with a constant initial rate. Tamm *et al.* reported that the depurination rate of calf thymus DNA was quite slow during the first several hours and became faster subsequently [16]. The possible reason causing this unexpected slowness is the precipitation of long DNA under strong acidic conditions. The rate of depurination slowed down because the reaction became heterogeneous. With the progress of depurination, the precipitation of DNA may have been dissolved gradually, so that the rate of depurination recovered and showed the dynamics of a first-order reaction.

Obviously, it is difficult to observe depurination directly in physiological conditions due to its extremely slow rate. Although some researchers have concluded that depurination occurred *in vivo* by measuring depurination at neutral pH and high temperature above 70°C [10], [19], there has been no report on detecting depurination directly at physiological conditions until now. In this study, the depurination rate constant at physiological conditions (pH 7.1, 37°C) was obtained for the first time by lengthening the reaction time to several months, and the rate constant (*k*) of N30 was obtained as 2.4×10^−10 ^s^−1^ at 37°C and pH 7.1 (Table 1). The rate constant was 1.5×10^−8 ^s^−1^ at 70°C and pH 7.4, which is in agreement with the results obtained by Lindahl *et al*. (1.6×10^−8 ^s^−1^ for ssDNA) [10].

Regarding the effect of DNA state (ds or ss) on depurination, Lindahl *et al*. investigated the depurination of thermal denatured *Bacillus subtilis* DNA at pH 7.4 and 70°C to clarify the depurination rate of ssDNA [10]. Their results about the difference between ssDNA and dsDNA (4-fold at pH 7.4) is higher than our results in Figure 6 (approximately twice at pH 5.1). In our experiments, several kinds of DNA were applied in depurination and the differences between ssDNA and dsDNA at pH 5.1 were basically similar, indicating that the potential effect of sequences was not the factor causing the lower difference. Therefore, the lower difference between dsDNA and ssDNA in our study is attributed to the low pH value we used. Compared with that under neutral conditions, dsDNA prefers to dissociate under acidic conditions [10], and the dissociation may become more easily once its depurination happens.

Both our data and the reported data showed that depurination of ssDNA were several times faster than that for dsDNA. Compared with the effect of duplex formation on deamination of cytosine (the rate of dsDNA is only 0.5%–0.7% of that of ssDNA) [24], the protection of duplex formation against depurination was not as conclusive. On the other hand, most genomic DNA in cell nuleuses wraps on histones, rather than in a naked state as we used for depurination. We found that the depurination rate reduced by 95% after adding spermine, a polycation compound similar as histones (Figure S4). The interaction of sperimine and histones with DNA may protect it from depurinating, which is the case *in vivo*. During replication and transcription, therefore, single-stranded fragments formed in genomic DNA may suffer from higher-frequency depurination, leading to the possibility of mutation.

**Figure 7:**
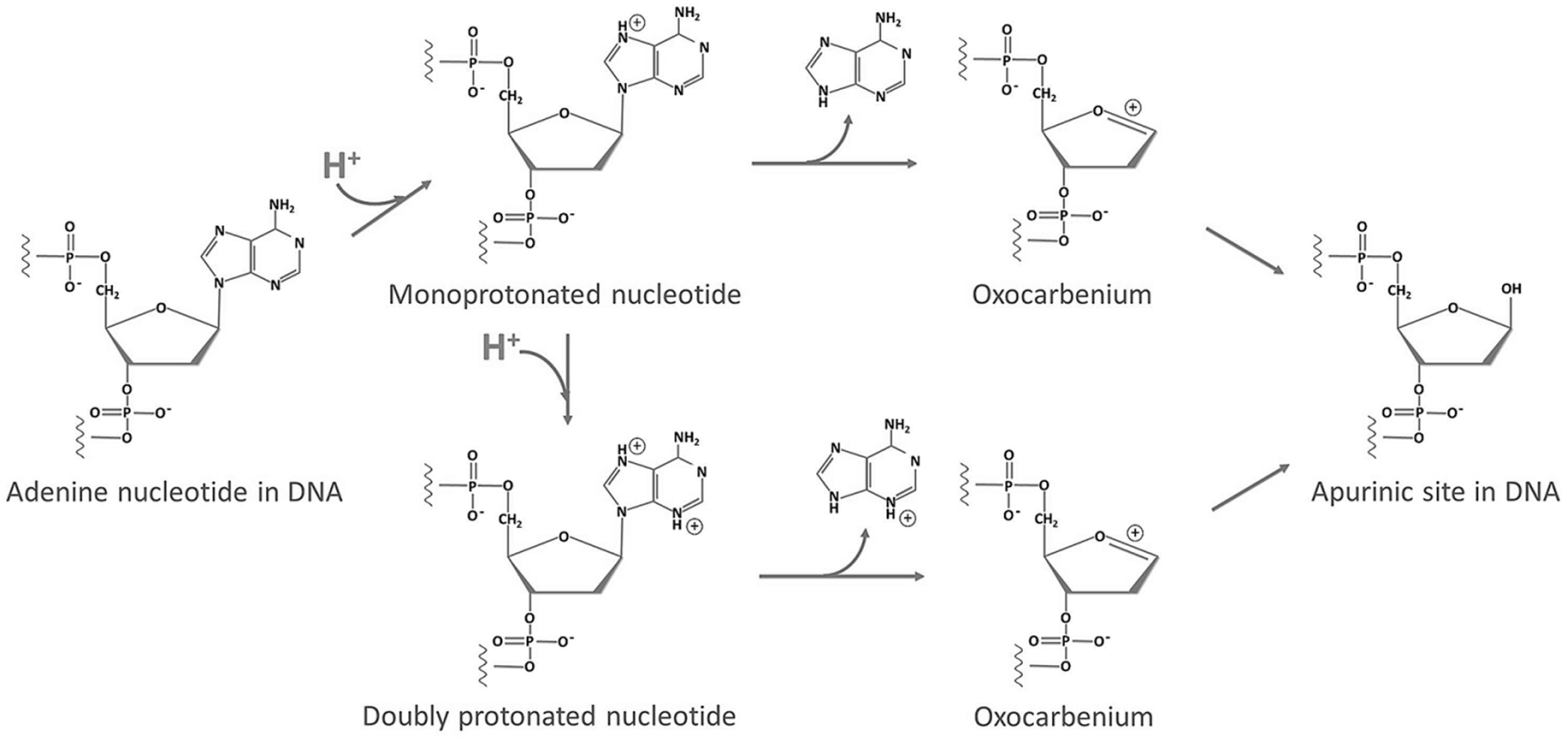
Protonation mechanism of depurination. Adenine is monoprotonated on N7 under acidic conditions, finally forming a neutral adenine and a deoxyribose as the end products. As the pH lowers, the double protonation process occurs by the attack of excessive H^+^ on N3.

The current accepted mechanism of acid-catalyzed depurination is illustrated in Figure 7 [25]–[29]. Take adenine as an example, the attack of H^+^ at N7 of adenine leads to the formation of a monoprotonated intermediate, which causes a series of charge redistribution. Then, an oxocarbenium ion and a neutral adenine form after cleavage of the glycosidic bond. The oxocarbenium ion reacts with a H_2_O molecule, giving a deoxyribose and a neutral adenine as the end products. Under strong acidic conditions, almost all purines are monoprotonated, but the depurination rates still increased linearly with the decline of pH values (Figure 2), indicating that the presence of double protonation may accelerate depurination [13]. During the double protonation of adenine, hydrogen ions are prone to attack purines at N3 [20], [28], and the final products are a deoxyribose and a positive charged adenine (Figure 7). It has been reported that protonation on N7 can greatly lower the energetics of the transition state (∼10 kcal/mol), while the second protonation on N3 promotes depurination by causing bases to adopt a synperiplanar or strongly chiral structure, rather than dramatically decreasing the transition state energy [30], [31]. Accordingly, no large difference between the activation energy (*E_a_*) of depurination at pH 1.0–2.0 (double protonation) and that at pH 4.1 (monoprotonation) was observed (Figure 3).

The effects of pH, sequences and salts on depurination can also be explained by the above mechanism. In terms of pH, the relationship between lg *k* and pH was not a simple straight line but a line with a salient point at pH approximately 2.5 (Figure 2). This result was basically in agreement with the results obtained by Hevesi *et al.* [18], and the salient point in their study was around pH 2. This phenomenon was most likely caused by different protonation styles of bases at pH below and above pH 2.5. The *pKa* values of guanosine monophosphate and adenosine monophosphate are 2.4 and 3.8 [32], respectively. In the case of low acid concentration (pH>2.5), only a part of purines were monoprotonated. Accordingly, with the decrease of pH values, the number of protonated purines increased and the depurination rate grew linearly. Under higher acidities (pH<2.5), most of the purines and cytosines were monoprotonated or even doubly protonated, so that the number of positive charges on bases increased sharply. Therefore the massive positive charges may suppress further attack of protons on other neighboring neutral purines. As a consequence, below pH 2.5, the growth rate of lg *k* with the decrease of pH, represented by the absolute value of the slopes of regression line in the profile, was smaller than those above pH 2.5 (Figure 2).

In a similar way, the sequence effect on depurination may also be attributed to the protonation of bases. In terms of the repetitive sequences made up of adenine, cytosine and guanine nucleotides, such as A30 and GC15, adenine, cytosine and guanine are prone to be protonated [32] and carry positive charges under acidic conditions. High density of positive charges on these bases may restrain further protonation of adjacent neutral purines, resulting in the slow depurination of these sequences. On the other hand, in the repetitive sequences such as AT15 and GT15, thymine is unable to be protonated under acidic conditions [32], the density of positive charges decreases sharply due to the presence of neutral thymine. As a result, in these sequences, low-density positive charges had little influence on the protonation of adjacent purines, and the depurination rates were faster than other repetitive sequences without thymine. In the case of low pH, most of purines and cytosines were protonated resulting in the extreme high density of positive charges, which strongly restrained the depurination rates. However, at a relative high pH value, due to the low extent of protonation, the inhibition effect of positive charged bases on depurination was weakened. Therefore, the dependence of depurination rates on DNA sequences decreased gradually with an increase in pH (Figure 5). Additionally, the stacking of purines may also have effect on depurination rates. However, with a decrease in pH values, the stacking interaction was weakened [33], indicating that the stacking of purines is not the main reason for difference among depurination rates of various sequences.

The suppression of depurination by metal ions (Figure 4) could be explained by the interaction with the phosphate groups of DNA [34]. Because the negative charges on phosphate groups are beneficial for depurination by accelerating the protonation of purines, metal cations may neutralize the negative charges and subsequently suppress depurination. This can also be demonstrated by the dependence of depurination from nucleoside derivatives (deoxyadenosine, dAMP, dADP and dATP) on the concentration of salts at pH 1.4 and 37°C. Salts had no suppression on the depurination of deoxyadenosine; as the number of phosphate groups on nucleotides increased, salts showed an increasingly suppressive effect on depurination (Figure S5). Although the influence of salts on depurination has been roughly investigated in the past [10], [19], the significant dependence of suppression degree of salts on pH values was found for the first time. In the case of pH 5.9, much higher than the *pKa* of phosphate groups on DNA (∼1.5), almost all the phosphate groups were ionized and carried negative charges. Therefore metal cations had a strong tendency to interact with them, and showed significant suppression on depurination (Figs. 4c and 4d). However, under the condition of pH 1.4, close to the *pKa* of phosphate groups, over 50% of the phosphate groups were not ionized. Accordingly, the effect of salt concentration on depurination became insignificant in the case of pH 1.4 (Figs. 4a and 4b).

Based on our results, two equations were obtained to predict the rate constant of depurination. We have estimated 49 rate constants for various types of DNA at various conditions, including M13 ssDNA, and the errors between predicted and experimental data were within 32%, which suggests that our predicting equations can estimate the rates of depurination successfully. Additionally, other factors affecting depurination, such as salts, duplex state and DNA sequence, should also be considered, and the predicted values should be adjusted slightly based on actual conditions and substrates.

In conclusion, non-enzymatic depurination of ODNs obeys a model of typical first-order kinetics. The logarithms of the rate constants (lg *k*) of depurination increase with a decrease in pH, and show a pseudo linear progression with an infection point at approximately pH 2.5. Besides, salts have strong protection against depurination and the suppression degree of salts depends greatly on pH values. In addition to the external factors, some internal aspects also affect depurination to different degrees. The remarkable effect of DNA sequences on depurination was found for the first time. DNA sequences without thymine are much less reactive than others, and depurination of poly (dA), such as A30 used here, is extremely low, especially at lower pH values. The rate for ssDNA is a little higher than that for dsDNA. Finally, the prediction of depurination under various pH values and temperature was realized by our equations. Our study may be useful to rectify an incorrect understanding of depurination and provides basic data for physiological activities and diseases involving gene mutagenesis, nucleic acid digestion and the possible incentive of cancer.

## Supporting Information

S1 Fig **HPLC chromatogram for free nucleobases.** (a) Five types of standard bases were mixed and injected in HPLC. (b) The mixture of N30 and uracil (internal standard) was injected in HPLC after incubation in 50 mM sodium phosphate buffer (pH 1.6) for 2 h at 37°C. Peak 1: Cytosine; Peak 2: Uracil; Peak 3: Guanine; Peak 4: Thymine; Peak 5: Adenine; Peak 6: DNA substrates.
(DOC)

S2 Fig
**Time courses of non-enzymatic depurination.** Quantitative analysis of purines released from N30 at 37°C. (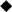) pH 5.1; (▪) pH 6.1; (•) pH 7.1. The percentages of depurination were the average values of released adenine and guanine. Reaction systems for depurination contained 50 mM sodium phosphate. Samples were prepared by collecting aliquots of the solution in each time period. For each point, three individual experiments were conducted and analyzed separately.(DOC)

S3 Fig **Melting curves of M13mp18 RF I DNA (M13 dsDNA) in 50 mM sodium phosphate solution (pH 5.1).** To minimize the depurination of M13 dsDNA during the *T*_m_ measurement at pH 5.1, only the heating curve (from 40°C to 98°C) was measured. The temperature ramp was 1.0°C/min. Melting temperature (*T*_m_) was obtained as 82.5°C. Accordingly, M13 dsDNA formed duplex well below 70°C.
(DOC)

S4 Fig **The suppression of spermine on depurination of N30.** The rate was analyzed in 50 mM sodium phosphate buffer (pH 3.0) at 37°C. The molar concentration of spermine was 75 folds as large as that of N30, i.e. the ratio of amine groups on spermine and phosphate groups on N30 (N/P) was 10∶1.
(DOC)

S5 Fig **The effect of salts on depurination from nucleotide derivatives.** (a) Depurination in the presence of various concentration of NaCl; (b) Depurination in the presence of various concentration of MgCl_2_. The substrates of depurination (to the final concentrate of 10 µM during depurination), including deoxyadenosine (dA), deoxyadenosine monophosphate (dAMP), deoxyadenosine diphosphate (dADP) and deoxyadenosine triphosphate (dATP), were incubated at 30°C for 45 min to limit the percentages of depurination to be less than 20%. The pH values of the solutions were adjusted to 1.4 (±0.1) with an aqueous solution of HCl. In each experiment, depurination rate constant in the pure HCl buffer was served as the reference.
(DOC)

S1 Table **Rate constants (10^5^×** ***k*** **, s^−1^) of some trinucleotide repeat sequences depurinated at pH 1.6 and 37°C.**
(DOC)
