# Supplementary material for: Non-Enzymatic Depurination of Nucleic Acids: Factors and Mechanisms
Source: PLoS One. 2014 Dec 29;9(12):e115950. doi: 10.1371/journal.pone.0115950 (PMC4278771; doi:10.1371/journal.pone.0115950)
Supplement: S1 Fig — HPLC chromatogram for free nucleobases. (a) Five types of standard bases were mixed and injected in HPLC. (b) The mixture of N30 and uracil (internal standard) was injected in HPLC after incubation in 50 mM sodium phosphate buffer (pH 1.6) for 2 h at 37°C. Peak 1: Cytosine; Peak 2: Uracil; Peak 3: Guanine; Peak 4: Thymine; Peak 5: Adenine; Peak 6: DNA substrates. (DOC) [file pone.0115950.s001.doc]

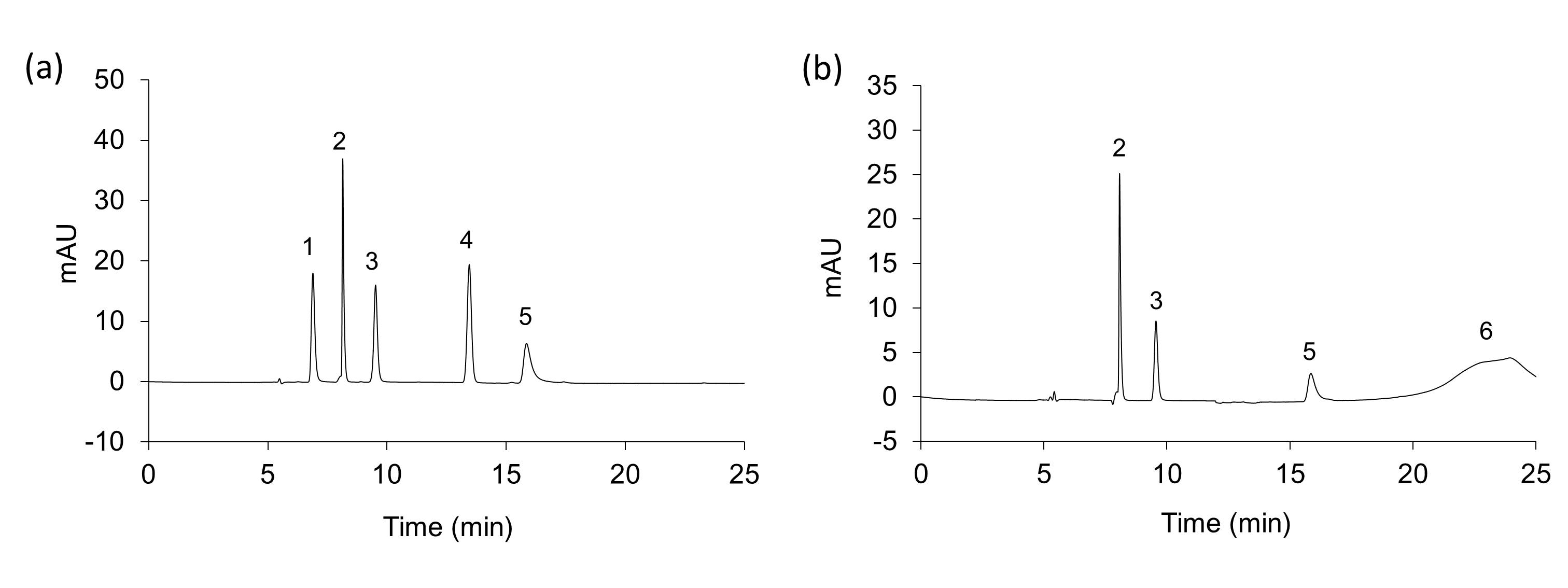


**Figure S1.** HPLC chromatogram for free nucleobases. (a) Five types of standard bases were mixed and injected in HPLC. (b) The mixture of N30 and uracil (internal standard) was injected in HPLC after incubation in 50 mM sodium phosphate buffer (pH 1.6) for 2 h at 37°C. Peak 1: Cytosine; Peak 2: Uracil; Peak 3: Guanine; Peak 4: Thymine; Peak 5: Adenine; Peak 6: DNA substrates.
