# Supplementary material for: Non-Enzymatic Depurination of Nucleic Acids: Factors and Mechanisms
Source: PLoS One. 2014 Dec 29;9(12):e115950. doi: 10.1371/journal.pone.0115950 (PMC4278771; doi:10.1371/journal.pone.0115950)
Supplement: S2 Fig — Time courses of non-enzymatic depurination. Quantitative analysis of purines released from N30 at 37°C. () pH 5.1; (▪) pH 6.1; (•) pH 7.1. The percentages of depurination were the average values of released adenine and guanine. Reaction systems for depurination contained 50 mM sodium phosphate. Samples were prepared by collecting aliquots of the solution in each time period. For each point, three individual experiments were conducted and analyzed separately. (DOC) [file pone.0115950.s002.doc]

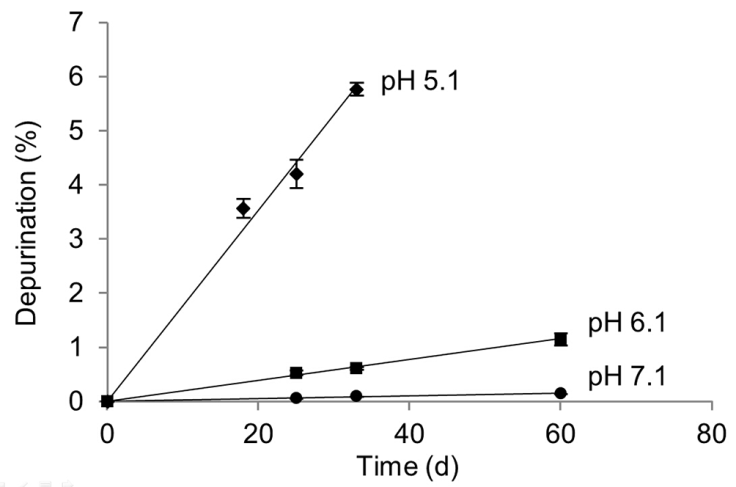


**Figure S2.** Time courses of non-enzymatic depurination. Quantitative analysis of purines released from N30 at 37°C. (◆) pH 5.1; (■) pH 6.1; (●) pH 7.1. The percentages of depurination were the average values of released adenine and guanine. Reaction systems for depurination contained 50 mM sodium phosphate. Samples were prepared by collecting aliquots of the solution in each time period. For each point, three individual experiments were conducted and analyzed separately.
