# Supplementary material for: Non-Enzymatic Depurination of Nucleic Acids: Factors and Mechanisms
Source: PLoS One. 2014 Dec 29;9(12):e115950. doi: 10.1371/journal.pone.0115950 (PMC4278771; doi:10.1371/journal.pone.0115950)
Supplement: S3 Fig — Melting curves of M13mp18 RF I DNA (M13 dsDNA) in 50 mM sodium phosphate solution (pH 5.1). To minimize the depurination of M13 dsDNA during the T m measurement at pH 5.1, only the heating curve (from 40°C to 98°C) was measured. The temperature ramp was 1.0°C/min. Melting temperature (T m) was obtained as 82.5°C. Accordingly, M13 dsDNA formed duplex well below 70°C. (DOC) [file pone.0115950.s003.doc]

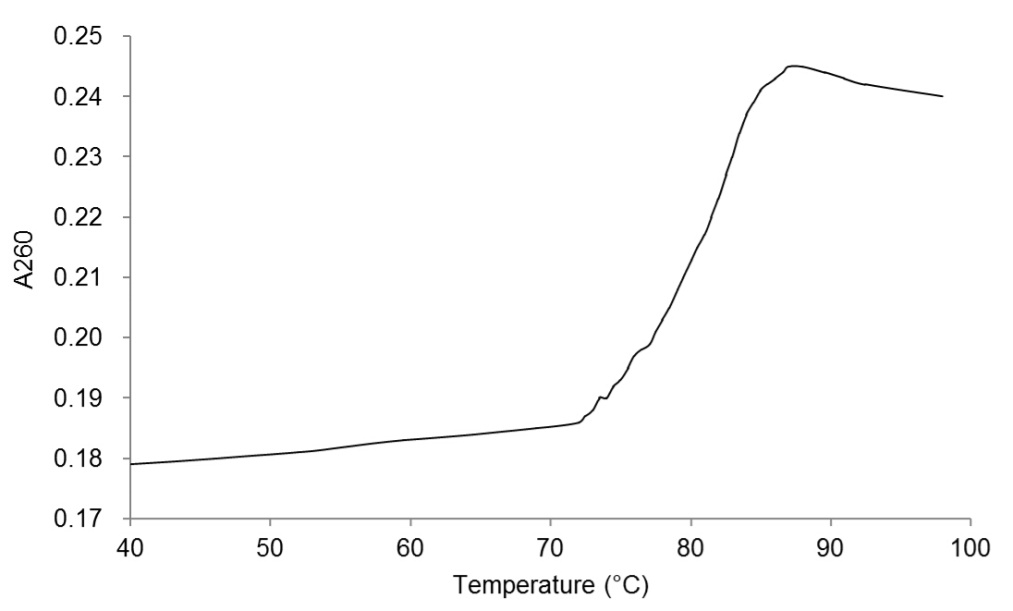


**Figure S3.** Melting curves of M13mp18 RF I DNA (M13 dsDNA) in 50 mM sodium phosphate solution (pH 5.1). To minimize the depurination of M13 dsDNA during the *T*_m_ measurement at pH 5.1, only the heating curve (from 40°C to 98°C) was measured. The temperature ramp was 1.0°C/min. Melting temperature (*T*_m_) was obtained as 82.5°C. Accordingly, M13 dsDNA formed duplex well below 70°C.
