# Supplementary material for: Non-Enzymatic Depurination of Nucleic Acids: Factors and Mechanisms
Source: PLoS One. 2014 Dec 29;9(12):e115950. doi: 10.1371/journal.pone.0115950 (PMC4278771; doi:10.1371/journal.pone.0115950)
Supplement: S4 Fig — The suppression of spermine on depurination of N30. The rate was analyzed in 50 mM sodium phosphate buffer (pH 3.0) at 37°C. The molar concentration of spermine was 75 folds as large as that of N30, i.e. the ratio of amine groups on spermine and phosphate groups on N30 (N/P) was 10∶1. (DOC) [file pone.0115950.s004.doc]

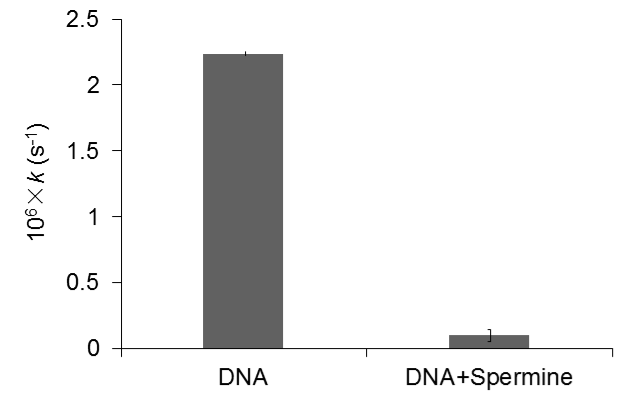


**Figure S4.** The suppression of spermine on depurination of N30. The rate was analyzed in 50 mM sodium phosphate buffer (pH 3.0) at 37°C. The molar concentration of spermine was 75 folds as large as that of N30, *i.e.* the ratio of amine groups on spermine and phosphate groups on N30 (N/P) was 10:1.

The depurination of N30 with spermine was much slower than that of pure N30. The rate constant of pure N30 was 1.1×10^-6^, 20-fold faster than that of N30 with spermine. The rate constant of N30 reduced by 95% after spermine was added to the system of depurination.
