# Supplementary material for: Non-Enzymatic Depurination of Nucleic Acids: Factors and Mechanisms
Source: PLoS One. 2014 Dec 29;9(12):e115950. doi: 10.1371/journal.pone.0115950 (PMC4278771; doi:10.1371/journal.pone.0115950)
Supplement: S5 Fig — The effect of salts on depurination from nucleotide derivatives. (a) Depurination in the presence of various concentration of NaCl; (b) Depurination in the presence of various concentration of MgCl2. The substrates of depurination (to the final concentrate of 10 µM during depurination), including deoxyadenosine (dA), deoxyadenosine monophosphate (dAMP), deoxyadenosine diphosphate (dADP) and deoxyadenosine triphosphate (dATP), were incubated at 30°C for 45 min to limit the percentages of depurination to be less than 20%. The pH values of the solutions were adjusted to 1.4 (±0.1) with an aqueous solution of HCl. In each experiment, depurination rate constant in the pure HCl buffer was served as the reference. (DOC) [file pone.0115950.s005.doc]

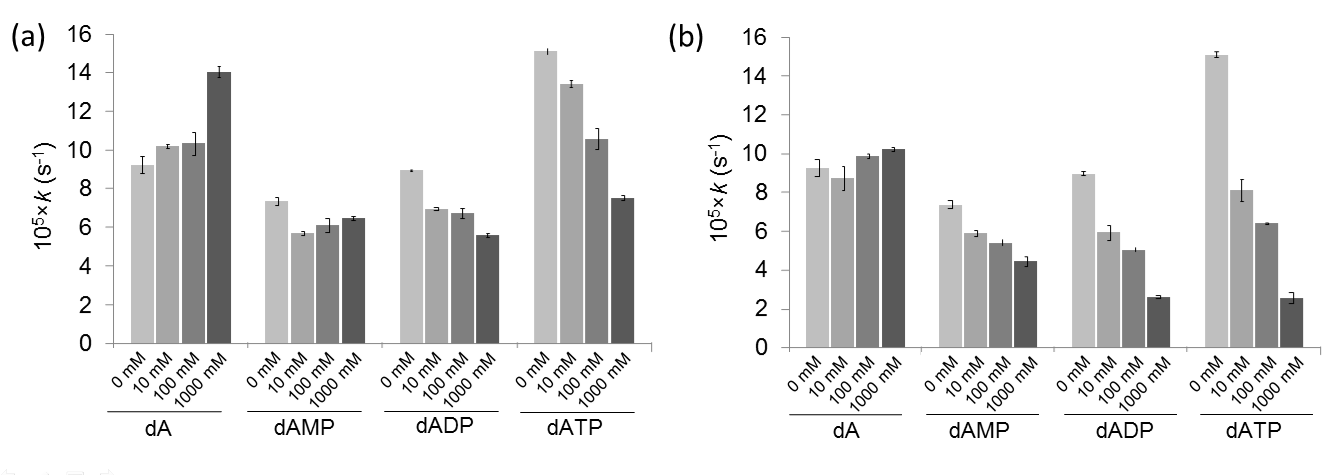


**Figure S5.** The effect of salts on depurination from nucleotide derivatives. (a) Depurination in the presence of various concentration of NaCl; (b) Depurination in the presence of various concentration of MgCl_2_. The substrates of depurination (to the final concentrate of 10 μM during depurination), including deoxyadenosine (dA), deoxyadenosine monophosphate (dAMP), deoxyadenosine diphosphate (dADP) and deoxyadenosine triphosphate (dATP), were incubated at 30°C for 45 min to limit the percentages of depurination to be less than 20%. The pH values of the solutions were adjusted to 1.4 (±0.1) with aqueous solution of HCl. In each experiment, depurination rate constant in the pure HCl buffer was served as the reference.

The suppression of depurination by salts increased with the number of phosphate residues, and the suppression by Mg^2+^ was stronger than Na^+^, which was consistent with the results of N30. It can be imaged that the effect of salts on depurination should become stronger at a higher pH (see Figure 4). In terms of deoxyadenosine (dA) which has no phosphate residues, its depurination was slightly accelerated by salts, rather than being suppressed.
