# Supplementary material for: Non-Enzymatic Depurination of Nucleic Acids: Factors and Mechanisms
Source: PLoS One. 2014 Dec 29;9(12):e115950. doi: 10.1371/journal.pone.0115950 (PMC4278771; doi:10.1371/journal.pone.0115950)
Supplement: S1 Table — Rate constants (105× k , s−1) of some trinucleotide repeat sequences depurinated at pH 1.6 and 37°C. (DOC) [file pone.0115950.s006.doc]

**Table S1.** Rate constants (10^5^×*k*, s^-1^) of some trinucleotide repeat sequences depurinated at pH 1.6 and 37°C.

| Name | Sequence (5′→3′) | Guanine  (10^5^×*k*, s^-1^) |  | Name | Sequence (5′→3′) | Adenine  (10^5^×*k*, s^-1^) |
| --- | --- | --- | --- | --- | --- | --- |
| TGT | TGTTGTTGTTGTTGTTGT | 3.95 |  | TAT | TATTATTATTATTATTAT | 3.98 |
| ATG | ATGATGATGATGATGATG | 3.86 |  | ATG | ATGATGATGATGATGATG | 3.78 |
| GGT | GGTGGTGGTGGTGGTGGT | 3.63 |  | AAT | AATAATAATAATAATAAT | 2.90 |
| AGG | AGGAGGAGGAGGAGGAGG | 2.19 |  | AGG | AGGAGGAGGAGGAGGAGG | 2.51 |
| CGG | CGGCGGCGGCGGCGGCGG | 1.88 |  | ACG | ACGACGACGACGACGACG | 1.69 |
| ACG | ACGACGACGACGACGACG | 1.68 |  | CAC | CACCACCACCACCACCAC | 1.43 |
| CGC | CGCCGCCGCCGCCGCCGC | 1.08 |  | AAC | AACAACAACAACAACAAC | 1.27 |
| AAG | AAGAAGAAGAAGAAGAAG | 0.96 |  | AAG | AAGAAGAAGAAGAAGAAG | 0.81 |

The order of depurination rates of these trinucleotide repetitive sequences indicates that sequences with thymine depurinated faster than other sequences, and depurination rates of sequences containing 2/3 thymine were faster than that of sequences containing 1/3 thymine. On the other hand, the depurination rates of sequences with more adenine were quite slow.
